# Dynamic orchestration of structural and immune cells in acute *Pseudomonas aeruginosa* pneumonia revealed by time-resolved single-cell and spatial transcriptomics

**DOI:** 10.3389/fimmu.2026.1874223

**Published:** 2026-09-08

**Authors:** Fuliang Zong, Yan Wang, Lingfei Hu, Lu Li, Dongsheng Zhou, Jin Zhao, Huiying Yang

**Affiliations:** State Key Laboratory of Pathogen and Biosecurity, Academy of Military Medical Sciences, Beijing, China

**Keywords:** circadian rhythm, host-directed therapy, ILC2, inflammatory fibroblasts, *Pseudomonas aeruginosa* pneumonia, single-cell RNA sequencing, spatial transcriptomics

## Abstract

**Background:**

*Pseudomonas aeruginosa* pneumonia triggers a complex, dynamic host response, yet the cellular coordination of inflammation and repair remains poorly defined.

**Methods:**

We performed time-resolved single-cell RNA sequencing (scRNA-seq) on murine lungs across five timepoints (0–96 h post-infection) and integrated these data with spatial transcriptomics (10x Visium) performed on matched tissue sections at key timepoints (0, 24, and 96 h), profiling a total of 41,452 high-quality cells.

**Results:**

We identified four neutrophil subsets, with the N3 population exhibiting potent antimicrobial activity. We traced the emergence of inflammatory fibroblasts during acute infection to Col13a1^+^ matrix fibroblasts, a process driven by a regulatory network centered on Stat1 and Nfkb1. Spatial transcriptomics with RCTD deconvolution revealed a 22.5% peak immune infiltration at 24 h, while COMMOT analysis uncovered 30-fold and 25-fold increases in *Ccl2–Ccr2 and Cxcl2–Cxcr2* spatial signaling, respectively, pinpointing chemokine-driven recruitment foci. Notably, spatial mapping distinguished tissue remodeling activity from cell-type abundance, identifying acute matrix remodeling zones dominated by *Timp1* rather than canonical fibroblast markers. Alveolar type 2 epithelial cells transitioned through three distinct states—homeostatic, inflammatory, and reparative. Notably, the circadian gene *Dbp* was specifically upregulated at 96 h, suggesting a role for circadian programs in resolution of pathology. Crucially, we uncovered a pivotal role for group 2 innate lymphoid cells (ILC2s): epithelial-derived IL-33 activated ILC2s via the ST2 receptor (*Il1rl1*) during acute infection (24 hpi), promoting a type 2 immune response that limits immunopathology.

**Conclusion:**

Our integrative time-resolved spatial transcriptomic atlas elucidates how structural and immune cells orchestrate the balance between bacterial clearance and tissue repair, highlighting novel targets for host-directed therapies in *P. aeruginosa* pneumonia.

## 1 Introduction

*Pseudomonas aeruginosa* represents a formidable opportunistic pathogen that causes severe and often life-threatening infections, particularly in immunocompromised individuals, patients with cystic fibrosis, and those requiring mechanical ventilation (1, 2). This Gram-negative bacterium establishes resilient infections in the respiratory tract due to its remarkable adaptability, extensive antibiotic resistance mechanisms, and sophisticated virulence factors that subvert host defenses (3, 4). Acute *P. aeruginosa* pneumonia is associated with mortality rates exceeding 40% in intensive care settings, highlighting the critical need for novel therapeutic approaches that target both the pathogen and the host response (5).

The mammalian lung has evolved intricate defense mechanisms to combat bacterial invaders like *P. aeruginosa*. Upon infection, a coordinated response involving both immune and structural cells is rapidly initiated to contain the pathogen while minimizing tissue damage (6). Neutrophils serve as first responders, deploying phagocytosis, neutrophil extracellular traps (NETs), and reactive oxygen species to eliminate bacteria (7). Alveolar macrophages contribute through pathogen recognition, phagocytosis, and cytokine-mediated recruitment of additional immune cells (8). Meanwhile, structural components of the lung—particularly epithelial cells and fibroblasts—actively participate in host defense through pathogen sensing, antimicrobial peptide production, and orchestration of inflammatory responses (9, 10). The successful resolution of *P. aeruginosa* infection thus hinges on the precise coordination between these cellular sentinels to achieve a delicate balance between pathogen eradication and the preservation of vital lung function.

Despite significant advances in understanding *P. aeruginosa* pathogenesis, our knowledge of the cellular dynamics during acute lung infection remains fragmented. Traditional bulk RNA sequencing approaches have provided valuable insights into overall transcriptional changes but fail to capture the heterogeneity within cell populations or identify rare but functionally important subsets (11). This limitation is particularly problematic in complex tissues like the lung, where diverse cell types interact dynamically during infection and resolution phases. Recent advances in single-cell RNA sequencing (scRNA-seq) technologies have revolutionized our ability to dissect cellular heterogeneity and identify rare or transitional cell states that are masked in bulk analyses (12). Complementing this, spatial transcriptomics technologies now enable the mapping of these transcriptional profiles directly onto intact tissue sections (13, 14), preserving the architectural context of cellular interactions. Together, this integrated single-cell and spatial multi-omics approach provides an unprecedented view of the lung’s host defense landscape, revealing not only which cell types respond to *P. aeruginosa*, but also where they are located, how they are organized into functional niches, and with whom they communicate during infection (15).

In the context of *P. aeruginosa* lung infection, critical gaps remain in our understanding of several fundamental processes (1) the cellular source of inflammatory fibroblasts that emerge during infection; (2) the heterogeneity within neutrophil populations and their specialized functions during bacterial clearance; (3) the molecular transitions of alveolar epithelial cells from homeostatic to activated and recovery states; and (4) the regulatory networks that coordinate the resolution of inflammation and tissue repair. Addressing these gaps is essential for developing targeted interventions that enhance bacterial clearance while preventing immunopathology.

This study leverages the power of time-resolved single-cell transcriptomics and spatial transcriptomics to construct a comprehensive cellular atlas of the murine lung during acute *P. aeruginosa* infection and recovery. By analyzing over 41,000 cells across five time points (0, 12, 24, 48, and 96 hours post-infection), we map the dynamic changes in cellular composition, gene expression programs, and intercellular communication networks throughout the injury-repair continuum (11). We identify *Col13a1*^+^ matrix fibroblasts as the primary precursors of inflammatory fibroblasts during infection, characterize functionally distinct neutrophil subsets critical for bacterial clearance, and discover a previously unrecognized circadian regulatory program involving *Dbp* and *Per3* that emerges during the resolution phase. In addition to myeloid and structural cells, group 2 innate lymphoid cells (ILC2s) have emerged as critical regulators of tissue homeostasis and repair during pulmonary infections. Activated by alarmins such as IL-33 released from damaged epithelial cells, ILC2s produce type 2 cytokines that dampen excessive inflammation and promote epithelial regeneration. In this study, we apply robust cell type decomposition (RCTD) to map 15 major and 24 fine-grained cell populations onto tissue coordinates at 0, 24, and 96 h post-infection, and employ spatial-constrained optimal transport (COMMOT) to quantify ligand–receptor signaling flows at anatomical resolution. This multi-layered spatial framework enables us to (1) localize immune infiltration dynamics to specific parenchymal regions (2), identify chemokine-driven recruitment foci, and (3) dissect the relationship between cell-type abundance and microenvironmental activation state—distinctions inaccessible to dissociation-based methods.

Our findings provide a foundational resource for understanding host-pathogen interactions in *P. aeruginosa pneumonia* and reveal novel cellular targets for therapeutic intervention. By defining the precise cellular players and molecular pathways involved in successful infection resolution, this work advances our ability to develop host-directed therapies that could complement traditional antimicrobial approaches in treating this challenging pathogen.

## 2 Methods

### 2.1 Animal model and ethics statement

All animal experiments were conducted in strict accordance with the Guide for the Care and Use of Laboratory Animals and approved by the Institutional Animal Care and Use Committee (IACUC) of the Academy of Military Medical Sciences (AMMS: approval number IACUC-IME-2021-031). Six to eight-week-old female C57BL/6J mice were purchased from Beijing Vital River Laboratory Animal Technology Co., Ltd. Mice were housed under specific pathogen-free conditions with a 12-hour light/dark cycle and free access to food and water. All efforts were made to minimize animal suffering and to reduce the number of animals used.

### 2.2 Bacterial strain and infection model

*The Pseudomonas aeruginosa* strain PAO1 was obtained from the Institute of Pathogen Biology, Chinese Academy of Medical Sciences. Bacteria were cultured in Luria-Bertani (LB) broth at 37 °C to mid-logarithmic phase, washed twice with phosphate-buffered saline (PBS), and resuspended to the desired concentration. Mice were anesthetized with isoflurane and infected via aerosol inhalation with 50 μL of PBS containing 1×10^6^ CFU of *P. aeruginosa* PAO1. Control mice (0 h timepoint) received aerosolized PBS only. At predetermined timepoints (0, 12, 24, 48, and 96 hours post-infection), three mice per group were euthanized by intraperitoneal injection of pentobarbital sodium (150 mg/kg) following subcutaneous heparinization (500 U/kg) to prevent intravascular coagulation (11).

### 2.3 Single-cell suspension preparation

Lungs were perfused via the right ventricle with cold PBS to remove circulating blood cells. The trachea was cannulated, and lungs were inflated with 1 mL of digestion solution containing collagenase IV (1 mg/mL), DNase I (20 μg/mL), and dispase (1 U/mL) in RPMI-1640 medium. The lung tissue was carefully dissected, placed in 5 mL of the same digestion solution, and incubated at 37 °C with shaking at 200 rpm for 60 minutes. The digested tissue was mechanically dissociated using a plunger, filtered through a 70 μm cell strainer, and centrifuged at 350 × g for 5 minutes at 4 °C. Red blood cells were lysed using ACK lysis buffer for 4 minutes at room temperature, followed by addition of PBS containing 2% FBS to terminate lysis. After centrifugation, the cell pellet was resuspended in PBS, passed through a 200-mesh nylon filter, and counted using a hemocytometer with Trypan blue exclusion to assess viability. It should be noted that enzymatic digestion preferentially recovers immune cells, while epithelial and stromal cells are relatively underrepresented due to their higher susceptibility to dissociation-induced stress. This bias has been consistently observed in independent scRNA-seq studies of acute lung injury models, including our previous work in a ricin-induced diffuse alveolar damage model (16). Therefore, the cell proportions reported here reflect technical bias inherent to current scRNA-seq protocols and should not be interpreted as the absolute cellular composition of lung tissue.

### 2.4 Single-cell RNA sequencing

Equal volumes of single-cell suspensions from three mice per timepoint were pooled to minimize individual variations. Cell suspensions with >85% viability were submitted to Novogene (Beijing, China) for library preparation and sequencing using the 10x Genomics Chromium platform (v3 chemistry). Briefly, approximately 10,000 cells were captured per sample, and cDNA libraries were prepared according to the manufacturer’s protocol. Sequencing was performed on an Illumina NovaSeq 6000 platform with a target depth of 50,000 reads per cell.

### 2.5 scRNA-seq data preprocessing

Raw sequencing data were processed using Cell Ranger software (version 3.1.0; 10x Genomics) with default parameters to align reads to the mouse reference genome (mm10) and generate gene-cell count matrices. Ambient RNA contamination was removed using the SoupX (17) package (version 1.6.2) in R. Quality control filtering was applied to retain high-quality cells meeting the following criteria (1): number of unique genes detected between 600 and 6,000 (2); mitochondrial gene percentage <5% (3); ribosomal gene percentage >3% (4); red blood cell-related gene (Hbb-bs, Hba-a1, Hba-a2) percentage <0.1%. Potential doublets were identified and removed using the DoubletFinder package (version 2.0.3) with default parameters, resulting in a final dataset of **41,452** high-quality cells across all timepoints.

### 2.6 Data integration, dimensionality reduction, and clustering

Data analysis was primarily performed using R (version 4.1.2) and Seurat (18) (version 4.4.0). Individual sample datasets were integrated using the canonical correlation analysis (CCA) algorithm to remove batch effects. The integrated dataset was normalized using the “LogNormalize” method, and 2,000 highly variable genes were identified using the “vst” method. Principal component analysis (PCA) was performed on the scaled data, and the first 20 principal components were used for downstream analysis based on the elbow plot and JackStraw permutation test. Cell clustering was performed using the graph-based Louvain algorithm with a resolution parameter of 0.5. Non-linear dimensional reduction was performed using uniform manifold approximation and projection (UMAP) for visualization.

### 2.7 Cell type annotation and validation

Cell clusters were annotated based on canonical marker genes from the literature and the CellMarker 2.0 database (19). To validate cell type annotations, we employed two machine learning approaches: random forest and support vector machine (SVM). For each approach, the top 5 highly expressed genes for each annotated cell type were used as features. The dataset was randomly split into training (75%) and testing (25%) sets, and model performance was evaluated using accuracy, precision, and recall metrics.

### 2.8 Differential expression and functional enrichment analysis

Differentially expressed genes (DEGs) between clusters or conditions were identified using the Wilcoxon rank-sum test implemented in Seurat’s FindAllMarkers and FindMarkers functions, with thresholds of |log2FC| > 1 and adjusted p-value < 0.05. Gene Ontology (GO) and Kyoto Encyclopedia of Genes and Genomes (KEGG) pathway enrichment analyses were performed using the clusterProfiler (20) package (version 3.18.1) with p-value adjustment using the Benjamini-Hochberg method. Enrichment results with adjusted p-value < 0.05 were considered statistically significant. To evaluate inflammatory activity, we first applied the Ad Model Score for preliminary signature identification. The final inflammatory response score was then computed using the HALLMARK_INFLAMMATORY_RESPONSE gene set from MSigDB (**Supplementary Table 1**).

### 2.9 Transcription factor regulatory network analysis

Transcription factor (TF) regulatory networks were reconstructed using pySCENIC (21) (version 0.11.2), a Python implementation of the SCENIC workflow. This approach involves three sequential steps (1): co-expression module inference using GENIE3 (2), motif enrichment analysis using RcisTarget to identify direct TF targets, and (3) cell-specific TF activity scoring using AUCell. TF regulons with normalized enrichment scores >0.05 were considered active in a given cell population.

### 2.10 RNA velocity analysis

RNA velocity was calculated to infer the directionality of cellular state transitions using the scVelo (22) package (version 0.2.5). Spliced and unspliced mRNA counts were generated from the Cell Ranger output using velocyto.py. The dynamical model was employed to estimate transcription, splicing, and degradation rates for each gene. Velocity vectors were projected onto the UMAP embedding to visualize cellular trajectories. Confidence scores for velocity estimates were calculated using the cosine correlation between velocity vectors and actual transcriptional changes.

### 2.11 Spatial transcriptomics data processing and analysis

Spatial transcriptomics was performed on mouse lung tissue at 0 h (uninfected control), 24 h, and 96 h post PAO1 infection using the 10× Genomics Visium platform (one sample per time point). Raw sequencing data were processed with Space Ranger (10× Genomics), and downstream analysis was performed in Seurat v4.4.0 (18) and Scanpy (23). Each sample was normalized using the default LogNormalize method in Seurat, followed by principal component analysis (PCA) and uniform manifold approximation and projection (UMAP) for visualization.

To resolve cell type composition at single-spot resolution, we performed deconvolution using RCTD (Robust Cell Type Decomposition) (24) with the time-resolved scRNA-seq dataset generated in this study as the reference. Deconvolution was performed at two resolutions: a broad classification of 15 major cell types and a fine-grained classification of 24 subpopulations incorporating myeloid and neutrophil subsets. RCTD was run in doublet mode to account for spots containing multiple cell types. Cell type proportion maps were visualized as spot-level spatial scatter plots with continuous color scales.

To infer spatial cell–cell communication, we applied COMMOT (25) to model ligand–receptor signaling flows between spatial locations. Three ligand–receptor pairs were selected based on their relevance to infection-induced inflammation and tissue remodeling: Ccl2-Ccr2 (monocyte recruitment), Cxcl2-Cxcr2 (neutrophil chemotaxis), and Spp1-Cd44 (macrophage activation and tissue remodeling). Optimal transport scores were computed using the sum-sender metric.

To characterize spatially coordinated transcriptional programs, we performed spatial differential expression analysis comparing immune-rich versus immune-poor spots, defined as the top and bottom 30% of spots ranked by summed RCTD immune cell type weights. Differentially expressed genes were identified using the Wilcoxon rank-sum test with Bonferroni correction. In addition, spatial gene–cell type correlations were computed as Pearson correlation coefficients between individual gene expression levels and RCTD-derived cell type proportions per spot; the top 20 correlated genes per cell type were retained for downstream interpretation.

### 2.12 Statistical analysis

All statistical analyses were performed using R (version 4.1.2) and Python (version 3.9.7). For comparisons between two groups, Student’s t-test or Mann-Whitney U test was used depending on data normality. For multiple group comparisons, one-way ANOVA with Tukey’s *post-hoc* test or Kruskal-Wallis test with Dunn’s *post-hoc* test was applied. Statistical significance was defined as p < 0.05. All tests were two-tailed unless otherwise specified. Data are presented as mean ± standard deviation (SD) from biological replicates.

## 3 Results

### 3.1 Comprehensive cellular atlas of murine lungs during *P. aeruginosa* infection

**Figure 1:**
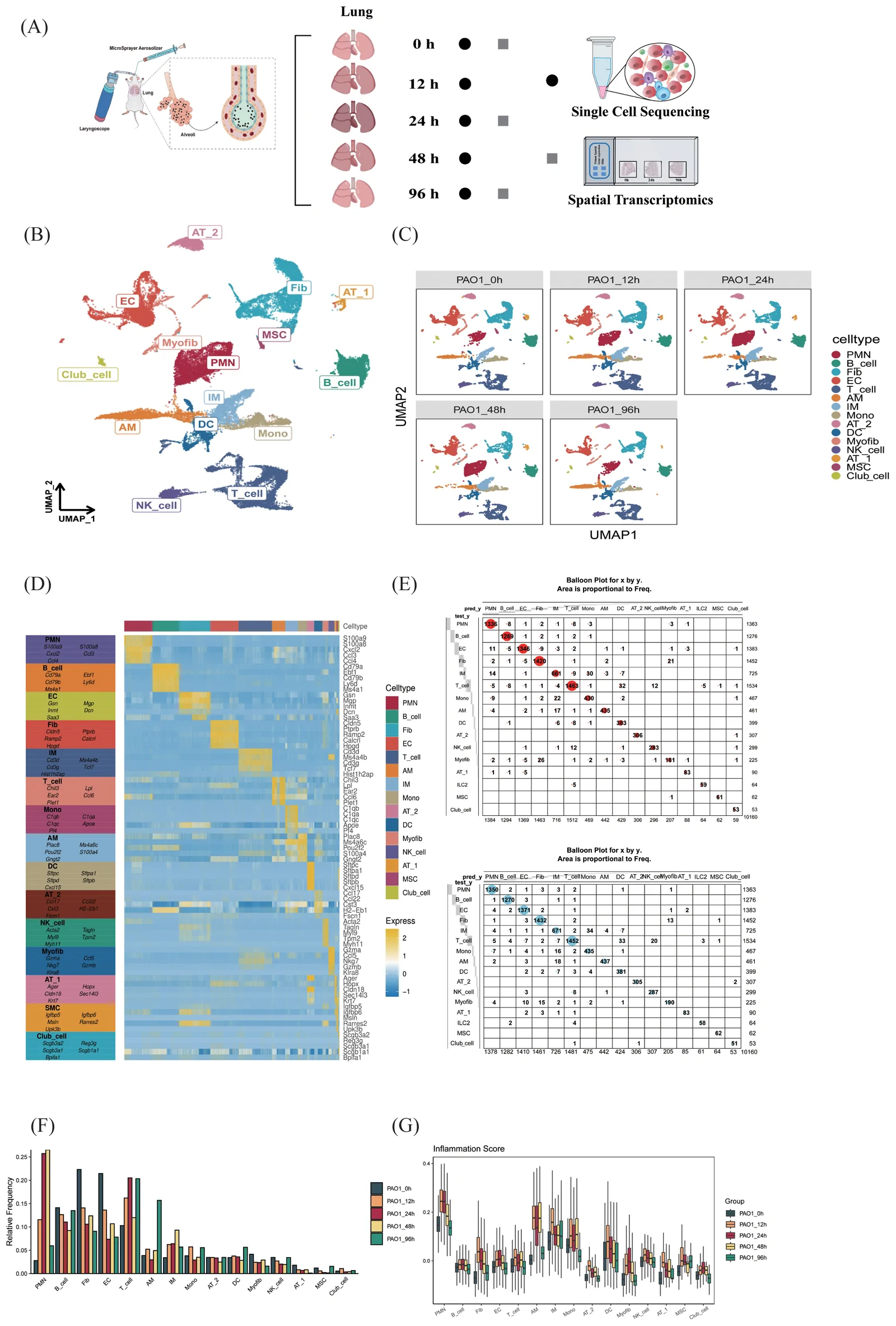
Comprehensive single-cell atlas of the murine lung during *Pseudomonas aeruginosa* infection. **(A)** Schematic overview of the integrated single-cell and spatial transcriptomics workflow. Lung tissues were collected at five timepoints (0, 12, 24, 48, and 96 hours post-infection) for scRNA-seq (10× Genomics Chromium), and matched tissue sections at 0, 24, and 96 h were processed for spatial transcriptomics (10× Genomics Visium). **(B)** UMAP embedding of 15 major cell types identified in murine lungs across all timepoints. Cell types are color-coded as indicated in the legend. **(C)** UMAP embeddings split by timepoint (0, 12, 24, 48, and 96 h post-infection), showing the temporal distribution of cells. **(D)** Heatmap showing the top five marker genes for each major cell type. Rows represent marker genes, and columns represent cell types. Color intensity indicates the average normalized expression level of each gene in the corresponding cell type. Only genes with significant differential expression (adjusted P < 0.05) are displayed. **(E)** Evaluation of cell type classification accuracy using support vector machine and random forest machine learning classifiers. **(F)** Stacked bar chart depicting temporal changes in cellular composition across the five timepoints. **(G)** Inflammatory scores of individual cell types across the five timepoints (0, 12, 24, 48, and 96 hours post-infection).

To investigate the cellular dynamics during acute *Pseudomonas aeruginosa* lung infection, we performed single-cell RNA sequencing (scRNA-seq) on lung tissues harvested from C57BL/6J mice at five timepoints: 0 (uninfected control), 12, 24, 48, and 96 hours post-infection (hpi) with *P. aeruginosa* PAO1 strain. To complement the dissociation-based transcriptomic profiling with spatial context, we additionally performed spatial transcriptomics using the 10× Genomics Visium platform on lung tissue sections at 0, 24, and 96 hpi (**Figure 1A**). These timepoints were strategically selected to capture the full spectrum of host responses, spanning the acute inflammatory phase (12–48 hpi) and the resolution/repair phase (96 hpi). After rigorous quality control, the scRNA-seq dataset comprised 41,452 high-quality cells with a median of 1344 genes and 3287 UMIs/counts per cell. The spatial transcriptomics dataset comprised 3334, 3497, and 3308 tissue-covered spots at 0, 24, and 96 hpi, respectively.

Unsupervised clustering and uniform manifold approximation and projection (UMAP) visualization revealed 15 major cell types in the murine lung (**Figures 1B, C**), including neutrophils (PMN), B cells, fibroblasts (Fib), endothelial cells (EC), T cells, alveolar macrophages (AM), interstitial macrophages (IM), monocytes (Mono), type II alveolar epithelial cells (AT2), dendritic cells (DC), myofibroblasts (Myo_fib), type I alveolar epithelial cells (AT1), natural killer cells (NK), smooth muscle cells (SMC), and Club cells. Each cell type exhibited canonical marker genes (**Figure 1D**): neutrophils expressed *S100a8* and *S100a9*, B cells expressed *Cd79a* and *Ms4a1*, endothelial cells expressed *Cldn5*, fibroblasts expressed *Mgp* and *Inmt*, while AT2 cells expressed *Sftpc* and *Sftpa1.*

To validate our cell type annotations, we employed two machine learning approaches-support vector machine (SVM) and random forest classification-using the top five highly expressed genes for each cell type as features (**Figure 1E**). Both methods achieved high accuracy (>92%), confirming the robustness of our cell type classification. Temporal analysis revealed dynamic changes in cellular composition during infection (**Figure 1F**). Notably, neutrophils showed the most dramatic expansion, increasing from 2.1% in uninfected lungs to 28.7% at 24 hpi before returning to baseline levels by 96 hpi, reflecting their central role in bacterial clearance (26).

To quantify inflammatory responses across cell types, we calculated an inflammatory gene signature score for each cell using the AddModuleScore function in Seurat (gene list provided in **Supplementary Table 1**). Neutrophils displayed the highest inflammatory scores during acute infection (12–48 hpi), which dramatically decreased by 96 hpi (**Figure 1G**). Myeloid cells (alveolar macrophages, interstitial macrophages, dendritic cells, and monocytes) showed similar but lower magnitude inflammatory responses. Remarkably, structural cells-including fibroblasts, myofibroblasts, and alveolar epithelial cells-also exhibited significant inflammatory activation during infection, demonstrating that non-immune cells actively participate in host defense (27).

### 3.2 Neutrophil heterogeneity reveals specialized subpopulations during infection

**Figure 2:**
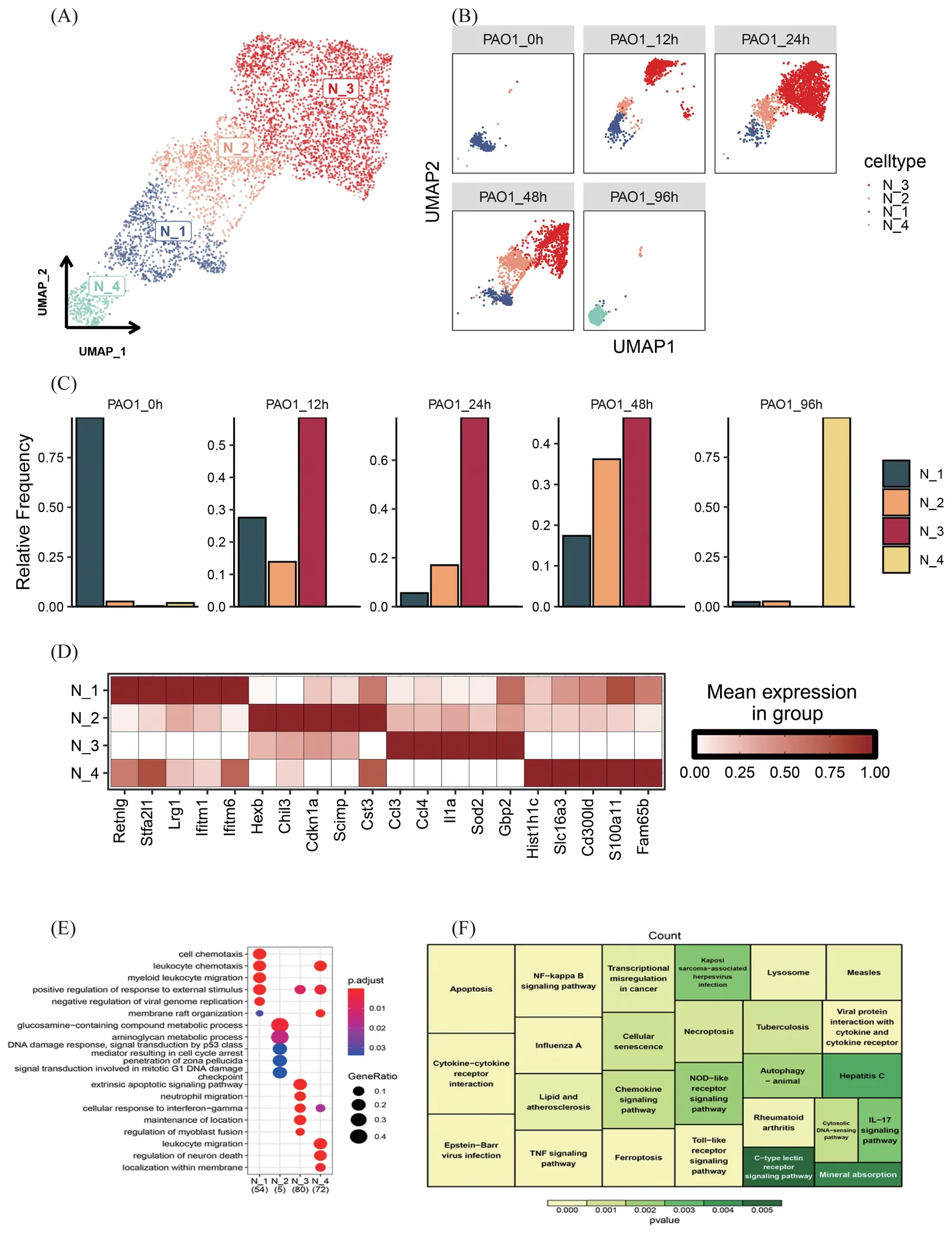
Neutrophil heterogeneity reveals specialized subpopulations during infection. **(A)** UMAP visualization of four neutrophil subpopulations identified by subclustering of 4,929 neutrophils, annotated as Homeostatic (N_1), Chil3^+^ Tissue-Remodeling (N_2), Inflammatory/Antimicrobial (N_3), and Activated/Metabolic (N_4) based on marker gene expression. **(B)** Temporal distribution of neutrophil subpopulations across five timepoints. **(C)** Bar plot shows the relative proportion (%) of each subpopulation (Homeostatic, Chil3^+^ Tissue-Remodeling, Inflammatory/Antimicrobial, and Activated/Metabolic). **(D)** Heatmap showing top 5 differentially expressed genes for each neutrophil subpopulation. **(E)** Gene Ontology (GO) enrichment analysis of biological processes for each neutrophil subpopulation. **(F)** KEGG pathway enrichment analysis specifically for the N_3 neutrophil subpopulation.

Given the central role of neutrophils in bacterial clearance, we performed subclustering of 4,929 neutrophils to identify functionally distinct subsets (**Figure 2A**). We identified four functionally distinct neutrophil subpopulations with distinct temporal distributions (**Figures 2B, C**): Homeostatic neutrophils (N_1), Chil3^+^ Tissue-Remodeling neutrophils (N_2), Inflammatory/Antimicrobial neutrophils (N_3), and Activated/Metabolic neutrophils (N_4). Homeostatic neutrophils predominated in uninfected lungs, Chil3^+^ Tissue-Remodeling neutrophils and Inflammatory/Antimicrobial neutrophils expanded during acute infection (12–48 hpi), and Activated/Metabolic neutrophils emerged during the resolution phase (96 hpi).

Each subset exhibited unique gene expression profiles (**Figure 2D**): Homeostatic neutrophils specifically expressed *Retnlg*, *Stfa2l1*, and *Ifitmt1/6*; Chil3^+^ Tissue-Remodeling neutrophils highly expressed *Hexb*, *Chil3*, and *Cdkn1a*; Inflammatory/Antimicrobial neutrophils uniquely upregulated *Ccl3*, *Ccl4*, *Il1a*, *Sod2*, and *Gbp2*; Activated/Metabolic neutrophils preferentially expressed *Hist1h1c*, *Slc16a3*, and *S100a11*. Notably, Inflammatory/Antimicrobial neutrophils expressed multiple antimicrobial effectors: *Ccl3* and *Ccl4* encode macrophage inflammatory proteins that recruit additional immune cells (28); *Il1a* encodes IL-1α, a central mediator of innate immunity; *Sod2* produces reactive oxygen species through respiratory burst; and *Gbp2* enhances defense against Gram-negative bacteria via pyroptosis (29). This specialized Inflammatory/Antimicrobial neutrophil subset likely represents the primary neutrophil population responsible for *P. aeruginosa* clearance.

Functional enrichment analysis revealed Homeostatic neutrophils were associated with “cell chemotaxis” and “leukocyte chemotaxis”, Chil3^+^ Tissue-Remodeling neutrophils with metabolic processes, and Inflammatory/Antimicrobial neutrophils with “extrinsic apoptotic signaling pathway” and “neutrophil migration” (**Figure 2E**). KEGG analysis showed Inflammatory/Antimicrobial neutrophils were significantly enriched in inflammatory pathways including “NF-kappaB signaling pathway”, “TNF signaling pathway,” and “Toll-like receptor signaling pathway” (**Figure 2F**).

### 3.3 Col13a1^+^ matrix fibroblasts are the primary source of inflammatory fibroblasts

**Figure 3:**
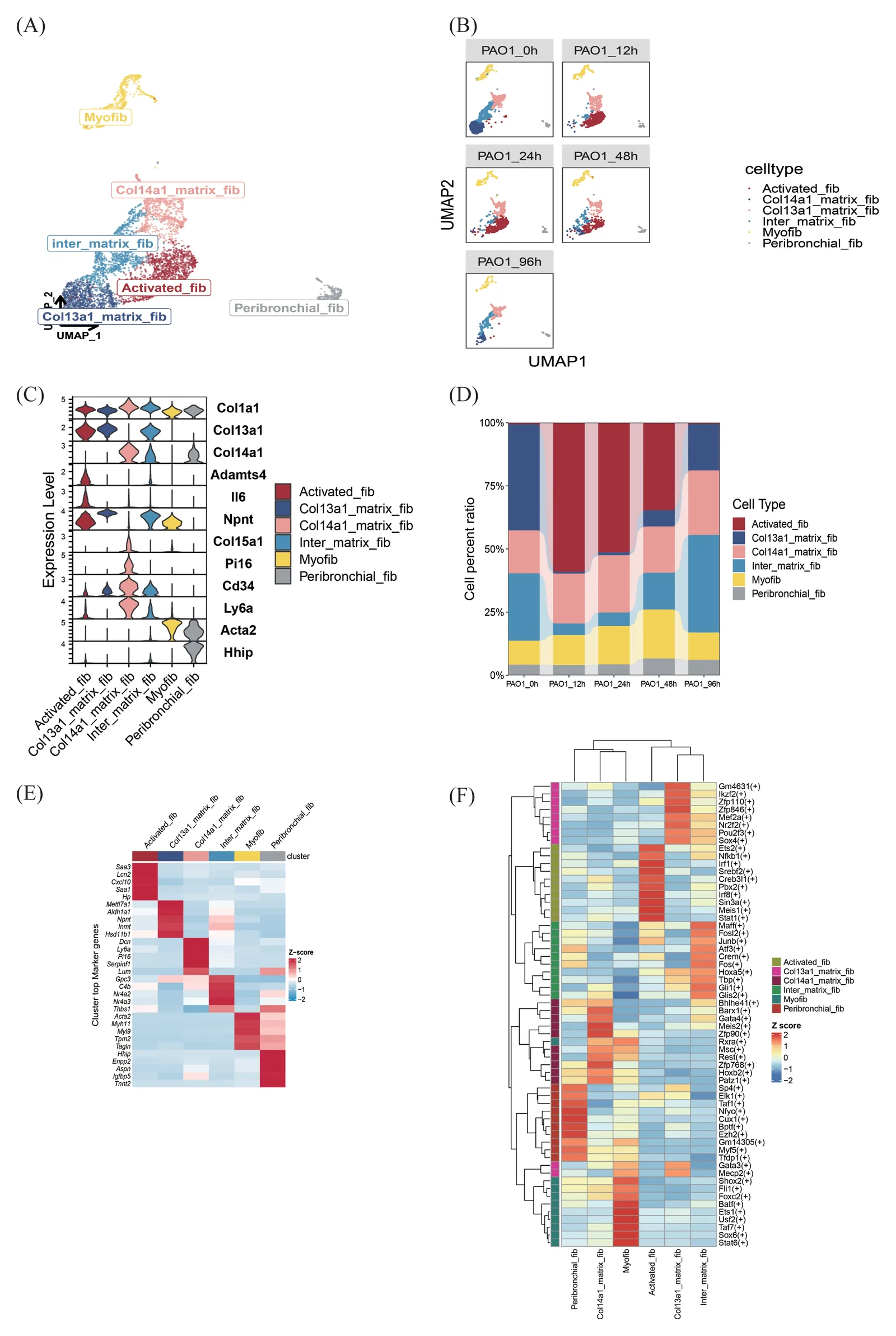
Col13a1^+^ matrix fibroblasts are the primary source of inflammatory fibroblasts. **(A)** UMAP visualization of six fibroblast subpopulations identified by subclustering of 5,461 fibroblasts. **(B)** UMAP embeddings of fibroblast subpopulations stratified by timepoint (0, 12, 24, 48, and 96 hours post-infection). **(C)** Feature plots showing expression of key marker genes across fibroblast subpopulations. **(D)** Bar chart showing the proportional changes of fibroblast subpopulations across five timepoints (0, 12, 24, 48, and 96 h post-infection). **(E)** Heatmap showing top differentially expressed genes in activated fibroblasts compared to other subpopulations. **(F)** Heatmap showing transcription factor expression across fibroblast subpopulations.

Subclustering of 5,461 fibroblasts identified six distinct subpopulations (**Figures 3A, B**): *Col13a1*^+^ matrix fibroblasts (Col13a1_matrix_fib), *Col14a1*^+^ matrix fibroblasts (Col14a1_matrix_fib), intermediate matrix fibroblasts co-expressing both markers (inter_matrix_fib), activated matrix fibroblasts (Activated_fib), myofibroblasts (Myo_fib), and peribronchial fibroblasts (Peribronchial_fib).

All fibroblast subpopulations expressed *Col11a1*, while exhibiting distinct marker profiles (**Figure 3C**). Activated fibroblasts specifically expressed *Col13a1*, *Adamts4*, *Il6*, and *Npnt*, but lacked *Col14a1*, *Col15a1*, and *Pi16*. In contrast, *Col14a1*^+^ matrix fibroblasts highly expressed *Col14a1*, *Col15a1*, *Pi16*, *Cd34*, and *Ly6a*, but lacked inflammatory markers.

Temporal analysis of cellular proportions showed activated fibroblasts were nearly absent in uninfected lungs but dramatically expanded at 12 hpi, gradually declining until returning to baseline by 96 hpi (**Figure 3D**). This decline correlated with a biphasic apoptotic signature peaking at 12 hpi (**Supplementary Figure 1A**), but was primarily attributable to massive immune cell infiltration diluting structural cell proportions, as activated fibroblasts maintained lower apoptotic and higher survival scores compared to homeostatic fibroblasts (**Supplementary Figures 1B, C**). This expansion inversely correlated with Col13a1^+^ matrix fibroblasts (decreasing from 34.7% to 8.2%), while Col14a1^+^ matrix fibroblast proportions remained stable throughout infection. These dynamic changes strongly suggest activated fibroblasts primarily derive from Col13a1^+^ matrix fibroblasts.

Activated fibroblasts specifically upregulated inflammatory mediators including *Saa3*, *Lcn2*, *Cxcl10*, and *Saa1* (**Figure 3E**). *Saa3* and *Saa1* belong to the serum amyloid protein family that regulates cytokine production during inflammation. *Lcn2* encodes lipocalin-2, which promotes intracellular lipid metabolism and inflammatory responses. *Cxcl10* encodes the chemokine CXCL10, which signals through CXCR3 to enhance neutrophil-mediated bacterial clearance. To spatially contextualize cell states identified by single-cell RNA-seq, we performed 10x Visium spatial transcriptomics on lung tissue at 0 h, 24 h, and 96 h post *P. aeruginosa* infection. Notably, spatial deconvolution using RCTD revealed a prominent population of activated fibroblasts specifically enriched in infected parenchymal regions at 24 hpi (**Supplementary Figure 2**), corroborating their existence as a distinct and spatially defined inflammatory cell state during acute infection.

Transcription factor analysis using pySCENIC revealed high regulatory potential for *Stat1*, *Nfkb1*, *Irf1*, and *Irf8* specifically in activated fibroblasts (**Figure 3F**). These factors form a core regulatory network driving inflammatory responses: STAT1 mediates immune cell differentiation, NF-κB orchestrates pro-inflammatory cytokine production, while IRF1/8 broadly regulate inflammatory gene expression. This transcriptional signature confirms the activated phenotype of these fibroblasts and identifies potential targets for modulating fibroblast-mediated inflammation.

### 3.4 Mononuclear phagocyte responses coordinate bacterial clearance and tissue repair

**Figure 4:**
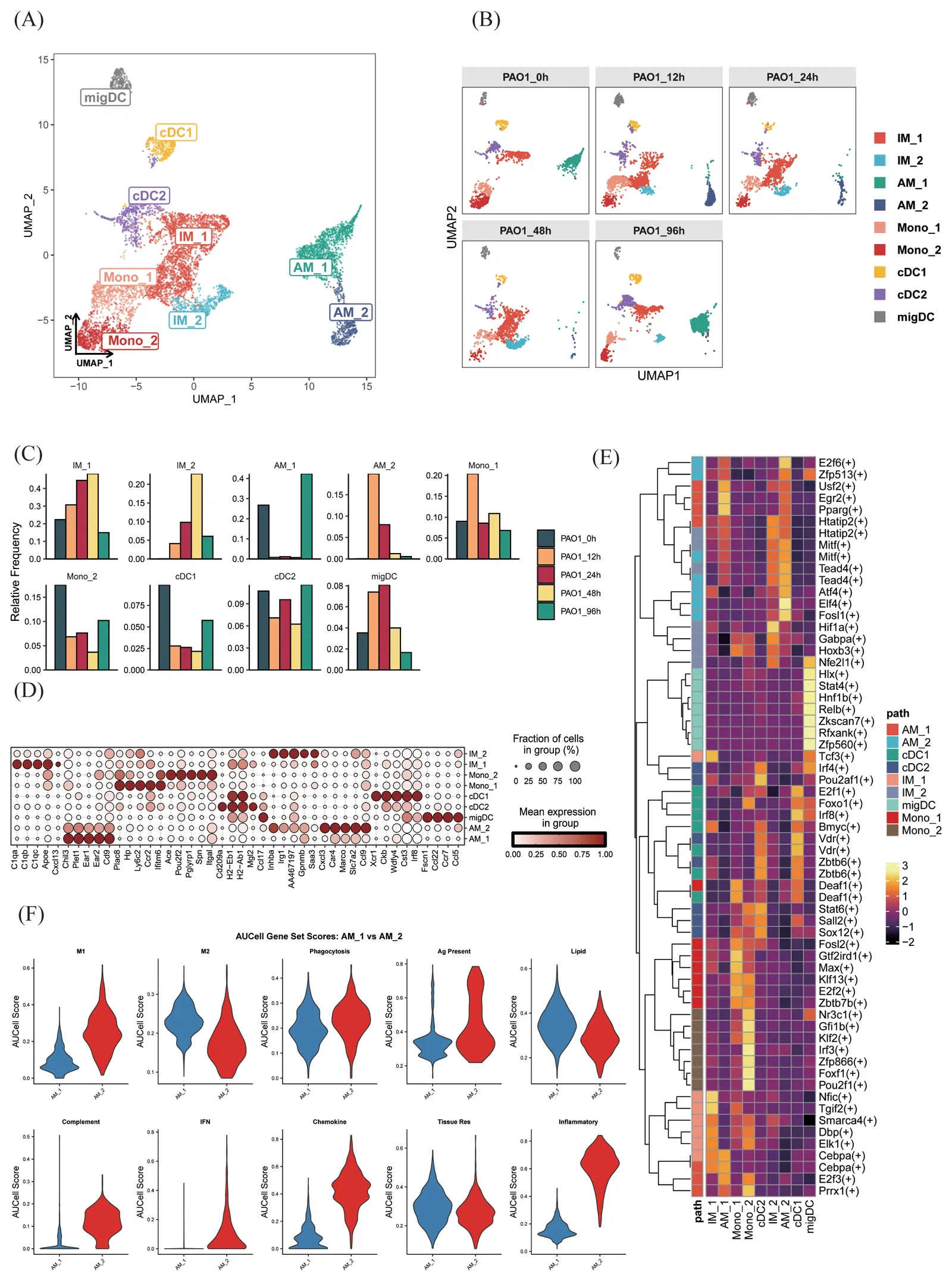
Mononuclear phagocyte responses coordinate bacterial clearance and tissue repair. **(A)** UMAP visualization of nine mononuclear phagocyte subpopulations identified by subclustering of 8,239 cells. **(B)** UMAP embeddings of Mononuclear phagocyte subpopulations stratified by timepoint (0, 12, 24, 48, and 96 hours post-infection). **(C)** Bar chart showing the proportion of each mononuclear phagocyte subpopulation. **(D)** Heatmap showing top 5 differentially expressed genes for each subpopulation. **(E)** Temporal changes in the proportions of mononuclear phagocyte subpopulations across five timepoints. **(F)** AUCell gene set scoring comparing functional signatures between AM_1 and AM_2 subpopulations. Pro-inflammatory scores include chemokine activity, complement activation, interferon response, and M1 polarization; anti-inflammatory/homeostatic scores include M2 polarization, lipid metabolism, and tissue-resident signatures. IM, interstitial macrophages; AM, alveolar macrophages; Mono, monocytes; DC, dendritic cells.

Mononuclear phagocytes play crucial roles in bacterial clearance, inflammation regulation, and tissue repair. Subclustering of 8,239 mononuclear phagocytes identified nine subpopulations (**Figures 4A, B**): two interstitial macrophage subsets (IM_1, IM_2), two alveolar macrophage subsets (AM_1, AM_2), two monocyte subsets (Mono1, Mono2), and three dendritic cell subsets (cDC1, cDC2, migDC).

IM_2 macrophages were nearly absent in uninfected lungs but expanded dramatically during infection (**Figure 4C**), suggesting a specific role in bacterial clearance. IM_2 cells specifically expressed *Irg1*, *AA467197*, *Gpnmb*, and *Saa3* (**Figure 4D**). *Irg1* encodes immune-responsive gene 1, which is highly expressed in inflammatory macrophages. *AA467197* encodes miR-147, which negatively regulates IL-6 and TNF expression following TLR4 activation, potentially limiting excessive inflammation. AM_2 macrophages predominated during acute infection and specifically expressed *Cxcl3*, *Car4*, *Marco*, *Slc7a2*, and *Ccl9*. *Cxcl3* encodes CXCL3, which recruits neutrophils to clear bacteria. *Marco* encodes the scavenger receptor MARCO, which recognizes various pathogens. *Ccl9* encodes MIP-1γ, which is upregulated by bacterial CpG DNA.

Transcription factor analysis revealed *Pparg* was likely active in homeostatic AM_1 macrophages (**Figure 4E**), consistent with its essential role in alveolar macrophage development. In contrast, AM_2 macrophages showed high regulatory potential for *Fosl1*, which negatively regulates type I interferon synthesis-suggesting a mechanism to balance immune activation during infection. To characterize the functional heterogeneity of alveolar macrophages during *P. aeruginosa* infection, we compared AM_1 and AM_2 subpopulations. AUCell gene set scoring revealed a clear functional dichotomy (**Figure 4F**): AM_2 exhibited significantly higher pro-inflammatory scores—including chemokine activity, complement activation, interferon response, and M1 polarization—whereas AM_1 was enriched for M2-like anti-inflammatory, lipid metabolism, and tissue-resident signatures. AM_2 was characterized by strong chemokine expression (*Cxcl3, Cxcl2, Cxcl9, Cxcl10, CCcl3–Ccl5*), interferon-stimulated genes (*Isg15, Gbp2/3/5, Irf7, Stat1*), and complement factors (*Cfb, C1qa/b/c*). Innate immune sensors (*Clec4e, Pycard, Il1b*) were also elevated, along with metabolic reprogramming (*Irg1/Acod1* detected in 82.2% of AM_2 vs. 1.3% of AM_1) and iron-sequestering genes (*Lcn2, Fth1, Hmox1*). In contrast, AM_1 maintained expression of tissue-resident markers (*Mrc1, Chil3, Sepp1*) and lipid metabolism genes (*Fabp4, Apoe, Lipa*), consistent with surfactant homeostasis. GO enrichment confirmed that AM_2-upregulated genes were enriched in LPS response and innate immune activation, while AM_1-upregulated genes were enriched in phagocytosis, lipid catabolism, and negative regulation of inflammation (**Supplementary Tables 2, 3**). These results suggest that AM_2 mediates pro-inflammatory bacterial clearance through chemokine-driven immune recruitment, complement activation, and interferon signaling, whereas AM_1 maintains tissue-resident homeostatic functions to support repair.

### 3.5 Alveolar epithelial cells undergo state transitions during infection and recovery

**Figure 5:**
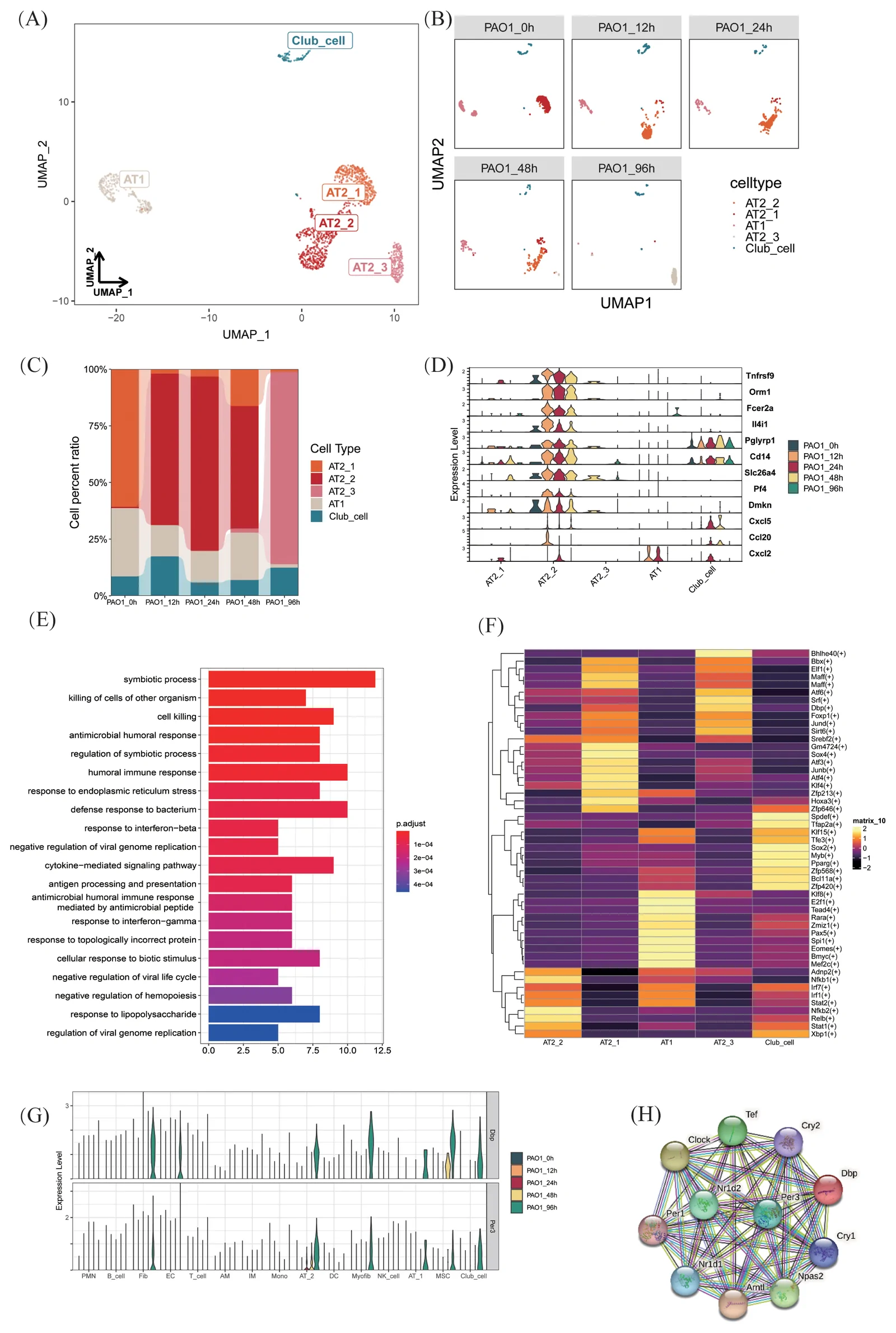
Alveolar epithelial cells undergo state transitions during infection and recovery. **(A)** UMAP visualization of five epithelial cell subpopulations identified by subclustering of 1,693 epithelial cells. **(B)** Temporal changes in the proportions of AT2 cell states across five timepoints. **(C)** Cell type composition of epithelial subpopulations across five time points post-infection, presented as stacked bar plots showing the proportion of each subpopulation relative to total epithelial cells. **(D)** Violin plots showing upregulated genes in the AT2_2 cells. **(E)** Gene Ontology **(GO)** enrichment analysis of biological processes for AT2_2-specific genes. **(F)** Heatmap showing transcription factor regulatory activities in AT2 subpopulations inferred by pySCENIC analysis. **(G)** Expression of Dbp and Per3 across all cell types. **(H)** Protein-protein interaction network showing predicted interactions between DBP and circadian regulators. AT1, alveolar type 1 epithelial cells; AT2, alveolar type 2 epithelial cells.

Alveolar epithelial cells serve as both physical barriers and active participants in host defense. Subclustering of 1,693 epithelial cells identified five subpopulations (**Figure 5A**): three type II alveolar epithelial cell states (AT2_1, AT2_2, AT2_3), type I alveolar epithelial cells (AT1), and Club cells. Temporal analysis revealed dramatic shifts in AT2 cell states during infection (**Figures 5B, C**): AT2_1 predominated in homeostasis, AT2_2 expanded during acute infection (12–48 hpi), and AT2_3 emerged during resolution (96 hpi).

AT2_2 cells, which dominate during bacterial clearance, specifically upregulated 65 genes compared to AT2_1/AT2_3 cells (**Figure 5D**), including *Tnfrsf9*, *Orm1*, *Fcer2a*, *Il4i1*, *Pglyrp1*, *Cxcl5*, *Ccl20*, and *Cxcl2*. Gene ontology analysis revealed enrichment in “cell killing”, “defense response to bacterium”, “response to interferon-beta”, and “response to lipopolysaccharide” (**Figure 5E**), indicating active participation in host defense. Transcription factor analysis predicted activation of *Nfkb1*, *Stat1*, *Irf1*, and *Irf2* in AT2_2 cells, forming a regulatory network that likely drives inflammatory gene expression (**Figure 5F**).

During resolution (96 hpi), AT2_3 cells specifically upregulated *Dbp*, which encodes the circadian transcription factor DBP (**Table 1**). This observation is consistent with our prior bulk RNA-seq analysis (11), which also revealed an increasing trend in Dbp expression at the 96 h time point (see **Supplementary Table 4**). Notably, *Dbp* was broadly upregulated in non-immune cells during resolution, including fibroblasts, myofibroblasts, AT1 cells, and smooth muscle cells (**Figure 5G**). Protein-protein interaction analysis predicted DBP interacts with PER3 (**Figure 5H**), a core circadian regulator. Consistent with this, *Per3* was also upregulated in our transcriptomic data at 96 hpi (**Supplementary Table 4**), suggesting circadian regulators may coordinate tissue repair during infection resolution.

**Table 1:** Differentially expressed genes in AT2_3 at 96 h post-infection versus 0 h.

Gene	avg_log2FC	pct.1	pct.2	p_val_adj	Direct
Dbp*	1.379581065	0.905	0.077	5.64E-89	up
*Gm42418*	2.733712657	1	1	4.23E-83	up
*Lars2*	1.922459364	0.995	0.828	2.34E-75	up
*Cyr61*	1.43002613	0.595	0.247	4.11E-19	up
*Scgb1a1*	-3.217523566	0.077	0.782	1.42E-54	down
*Hexim1*	-1.089335778	0.59	0.926	5.73E-45	down
*Ppp1r10*	-1.288809355	0.694	0.931	3.56E-43	down
*Slc38a2*	-1.019655034	0.842	0.971	3.68E-39	down
*Egr1*	-1.188013706	0.459	0.931	2.73E-35	down
*Klf4*	-1.087896372	0.221	0.745	2.50E-32	down
*Sox4*	-1.026985294	0.559	0.878	5.08E-30	down
*Nfkbia*	-1.116863435	0.698	0.894	1.95E-25	down
*Hspa1a*	-1.00318735	0.275	0.729	1.21E-20	down

Genes were identified using the Wilcoxon rank-sum test comparing AT2_2 at 96 hpi against 0 hpi. Columns: avg_log2FC, average log2 fold change (positive = upregulated at 96 hpi); pct.1, percentage of AT2 cells at 96 hpi expressing the gene; pct.2, percentage of AT2 cells at 0 hpi expressing the gene; p_val_adj, adjusted p-value (Bonferroni correction). Only genes with |log2FC| > 1 and adjusted p-value < 0.05 are shown. The asterisk (*) marks the genes that are further described in the main text (Dbp in Section 3.5).

### 3.6 ILC2 cells mediate type 2 immune responses during acute infection

**Figure 6:**
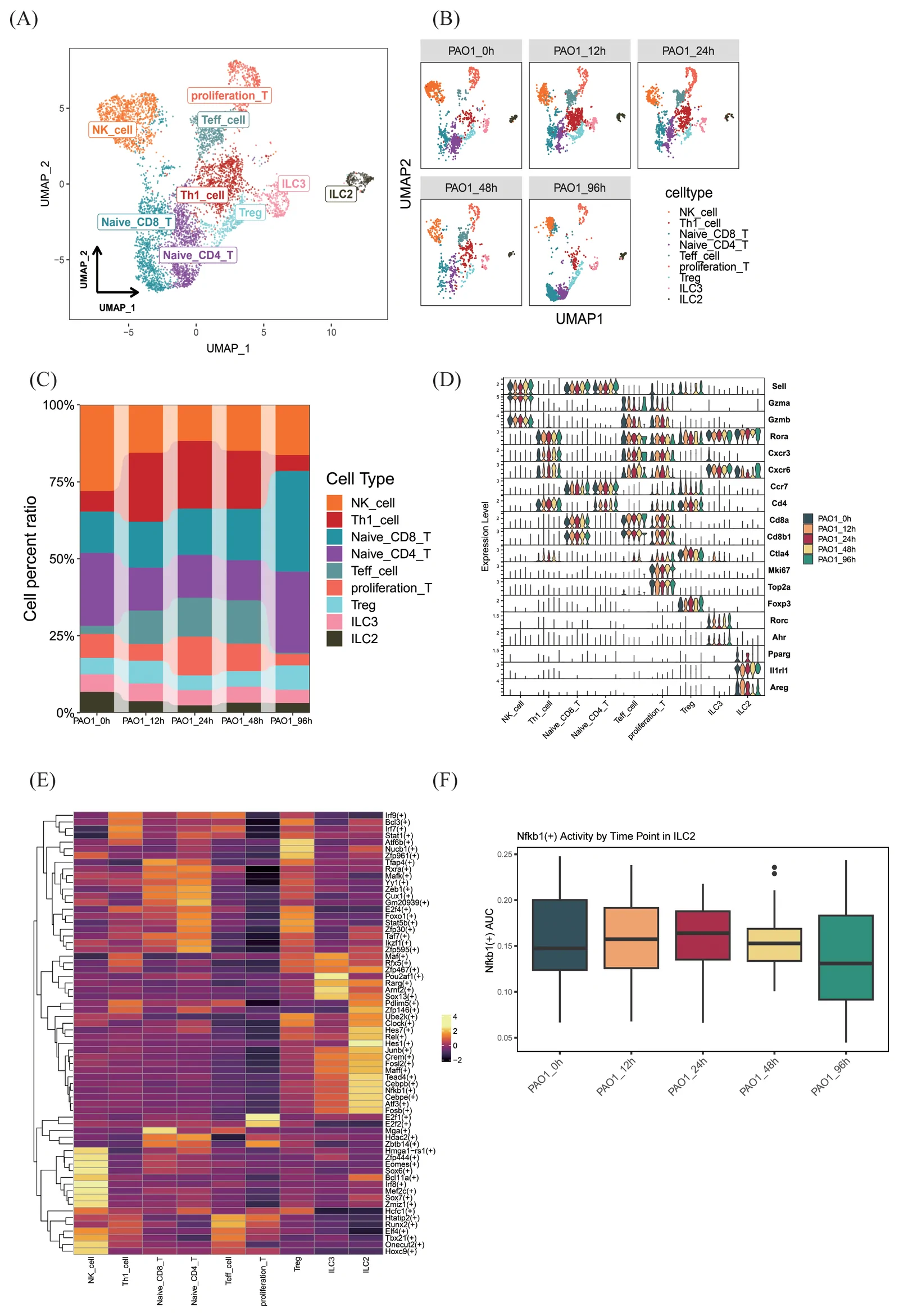
ILC2 cells mediate type 2 immune responses during resolution phase. **(A)** UMAP visualization of nine lymphocyte subpopulations identified by subclustering of 6,386 T and NK cells. **(B)** UMAP embeddings of T and NK cells subpopulations stratified by timepoint (0, 12, 24, 48, and 96 hours post-infection). **(C)** Bar chart showing the proportion of each lymphocyte subpopulation. **(D)** Violin plots showing expression distribution across cell types. **(E)** Heatmap showing transcription factor expression in lymphoid cell subsets. **(F)** Bar graph showing the activation level of ILC2s at different time points post-infection. NK, natural killer cells; ILC, innate lymphoid cells; Teff, effector T cells; Treg, regulatory T cells.

Re-clustering of 6,386 T and NK cells revealed nine distinct subpopulations, including group 2 innate lymphoid cells (ILC2s) (**Figures 6A, B**). Within the T/NK/ILC compartment, acute infection (12–24 hpi) was marked by a pronounced expansion of Th1 and T_eff cells, coupled with a contraction of NK and ILC2 cells, while proliferating T cells peaked at 24 hpi. During resolution (96 hpi), the landscape shifted dramatically: T_eff cells were nearly eliminated and Th1 cells declined, whereas naive CD8^+^ T cells became the dominant population, naive CD4^+^ T cells recovered (**Figure 6C**).

During acute *P. aeruginosa* infection, a specific subset of alveolar type 2 epithelial cells—designated AT2_2—showed selective upregulation of *Il33* (**Table 2**), which encodes the alarmin cytokine IL-33. IL-33 is released upon epithelial damage and signals through the ST2 receptor (30). Notably, ILC2s exhibited high expression of Il1rl1, the gene encoding ST2 (**Figure 6D**), positioning them as primary responders to IL-33 in this context. This IL-33/ST2 axis likely drives ILC2 activation during infection, triggering downstream NF-κB (encoded by *Nfkb1*; **Figure 6E**) and MAPK signaling pathways to promote the production of type 2 cytokines. Consistent with this, transcription factor activity analysis revealed dynamic regulation of NF-κB throughout the infection course: AUCell-based regulon activity scores for Nfkb1 showed an initial increase followed by a subsequent decline (**Figure 6F**), indicative of transient activation. Such temporal control may help balance effective pathogen clearance with the prevention of excessive inflammation, thereby supporting tissue repair. Together, these findings uncover a coordinated host defense network in which structural cells—notably fibroblasts and epithelial cells—actively collaborate with classical immune cells to orchestrate immune responses, resolve infection, and restore tissue homeostasis.

**Table 2:** Differentially expressed genes in AT2_2.

Gene	avg_log2FC	pct.1	pct.2	p_val_adj	Direct
*Lcn2*	4.56603347	0.998	0.965	1.60E-190	up	
*Tnfrsf9*	1.861702009	0.925	0.068	3.51E-183	up	
*Ly6i*	3.205903487	0.99	0.309	2.88E-181	up	
*C3*	2.377527187	0.998	0.775	1.53E-174	up	
*Orm1*	3.438221256	0.859	0.035	9.94E-169	up	
*Hp*	2.855514617	0.998	0.97	1.02E-168	up	
*Fth1*	3.238718319	1	1	1.59E-167	up	
*Tmem176a*	1.818620483	1	0.895	3.02E-167	up	
*Tmem176b*	1.597114542	1	0.955	6.66E-167	up	
*Slpi*	2.531096879	0.99	0.584	9.24E-164	up	
*Chil1*	1.726560536	0.998	0.99	8.70E-163	up	
*Sod2*	2.279678497	0.993	0.795	2.18E-162	up	
*Cyba*	2.295910168	0.989	0.725	1.27E-159	up	
*Sftpd*	1.121959039	0.998	0.998	1.35E-154	up	
*Fcer2a*	1.287255905	0.81	0.047	1.33E-148	up	
*Calr*	1.825333256	0.997	0.927	1.25E-144	up	
*Il4i1*	2.526042276	0.764	0.017	3.26E-143	up	
*Pglyrp1*	1.493673019	0.839	0.122	1.73E-140	up	
*Cd14*	2.50776169	0.907	0.327	8.15E-139	up	
*Gapdh*	1.142454057	0.998	0.992	3.31E-136	up	
*S100a11*	1.482084089	1	0.992	5.21E-136	up	
*Sec61g*	1.079519242	1	0.972	1.71E-134	up	
*Cd63*	1.204494178	1	0.988	9.83E-132	up	
*Slc26a4*	1.288575814	0.859	0.177	3.72E-129	up	
*Cd302*	1.178299033	0.957	0.446	1.69E-125	up	
*Clu*	1.840278607	0.992	0.818	3.74E-125	up	
*Ctps*	1.1120335	0.905	0.304	5.85E-123	up	
*H2afz*	1.446848103	1	0.955	8.54E-123	up	
*Cp*	1.289318459	0.743	0.063	2.28E-122	up	
*Psme2*	1.37035431	0.98	0.763	4.32E-122	up	
*Ifitm3*	2.744740157	0.877	0.341	8.72E-122	up	
*Psme1*	1.032369962	1	0.96	2.96E-121	up	
*Sdf2l1*	1.253824503	0.818	0.17	3.46E-119	up	
*Tapbp*	1.125568871	0.989	0.791	9.19E-119	up	
*Pdia3*	1.157833654	0.998	0.933	4.67E-116	up	
*Lrg1*	1.990568757	0.992	0.691	1.51E-115	up	
*Ctsc*	1.037337684	0.998	0.995	1.27E-114	up	
*Slc39a4*	1.105461931	0.936	0.467	1.61E-113	up	
*Manf*	1.467842654	0.967	0.746	4.02E-113	up	
*Bst2*	1.684720688	0.943	0.526	5.20E-106	up	
*Hspa5*	1.251065586	0.997	0.968	3.75E-105	up	
*Pdia6*	1.107555345	0.992	0.925	1.26E-102	up	
*Psmb8*	1.456817042	0.952	0.576	1.03E-101	up	
*Rps2*	1.066462649	1	1	9.58E-100	up	
*Pf4*	1.015038226	0.669	0.082	2.86E-93	up	
*Gm20186*	1.358796297	0.757	0.192	1.74E-90	up	
*Hsp90b1*	1.122596019	1	0.983	3.57E-89	up	
*Cd44*	1.127372209	0.966	0.689	3.45E-82	up	
*Icam1*	1.053956521	0.998	0.953	1.03E-81	up	
*Pigr*	1.154836146	0.669	0.127	5.63E-81	up	
*Ifitm2*	1.146285992	0.939	0.706	6.61E-80	up	
*Clip4*	1.01373222	0.974	0.775	2.89E-79	up	
*Il33*	1.410602902	0.985	0.898	1.93E-75	up	
*Tubb5*	1.001700874	0.911	0.566	2.80E-69	up	
*Tnip3*	1.027191217	0.498	0.042	1.47E-67	up	
*Dmkn*	1.136248956	0.646	0.16	5.05E-67	up	
*Nfkbia*	1.026710418	0.979	0.821	4.24E-62	up	
*Saa3*	4.868796954	0.425	0.022	1.16E-59	up	
*Gbp2*	1.102265926	0.466	0.068	5.60E-51	up	
*Cxcl5*	1.334748187	0.315	0.008	8.31E-43	up	
*Isg15*	1.236021681	0.534	0.157	1.25E-42	up	
*Ly6a*	1.356757615	0.757	0.456	2.28E-39	up	
*Ccl20*	2.706116136	0.272	0.003	4.49E-37	up	
*Cxcl2*	1.087920322	0.279	0.023	5.66E-31	up	
*Iigp1*	1.362582717	0.321	0.055	1.31E-28	up	

The genes were identified by comparing AT2_2 cells against AT2_1 and AT2_3 cells subpopulations using the Wilcoxon rank-sum test. Columns: avg_log2FC, average log2 fold change; pct.1 = percentage of AT2_2 cells expressing the gene; pct.2 = percentage of AT2_1/AT2_3 cells expressing the gene; p_val_adj, adjusted p-value (Bonferroni correction). Only genes with |log2FC| > 1 and adjusted p-value < 0.05 are shown.

### 3.7 Spatial transcriptomics reveals chemokine-driven immune cell recruitment

**Figure 7:**
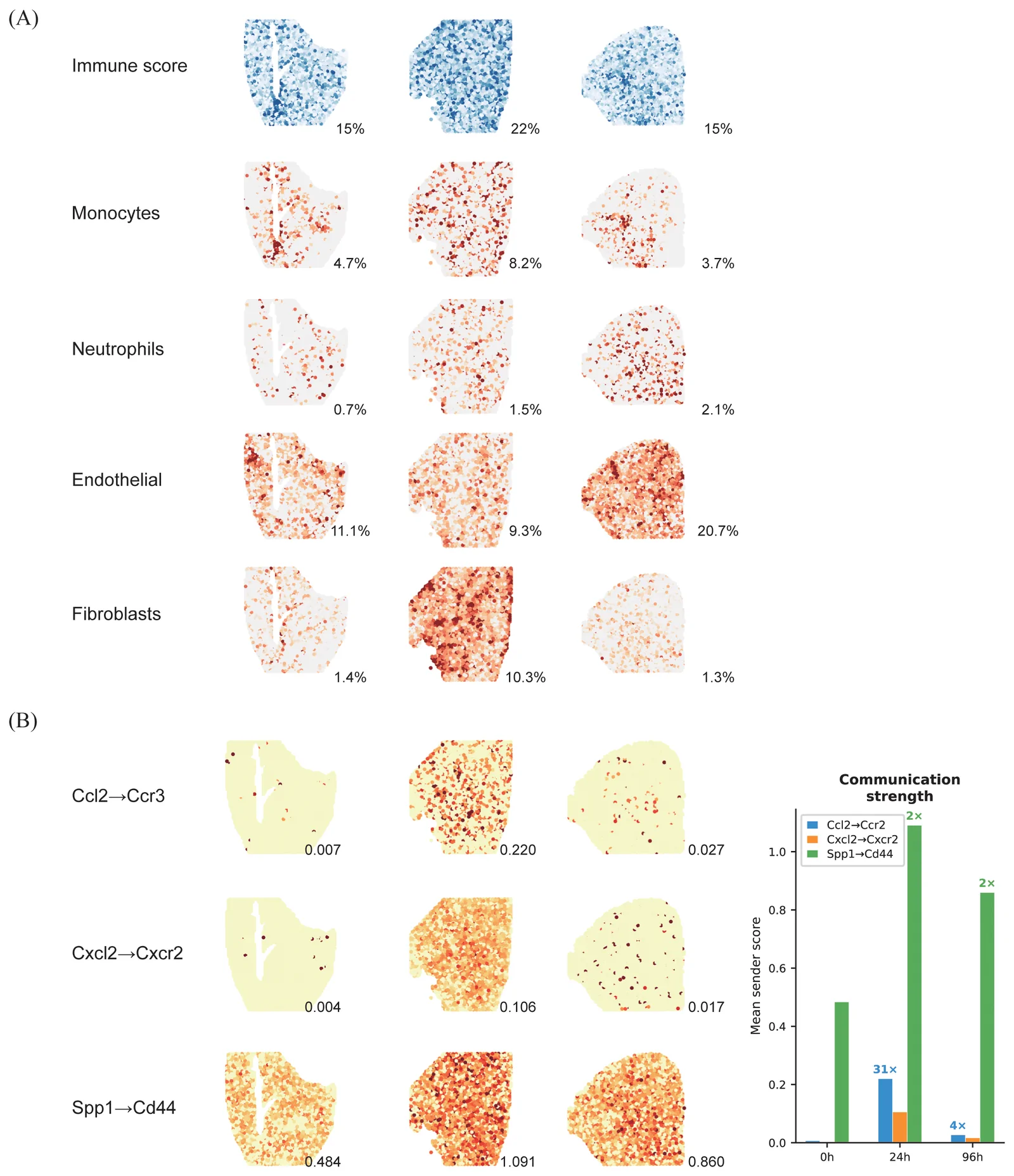
Spatial transcriptomic landscape of *P. aeruginosa*-infected lungs. **(A)** RCTD deconvolution maps showing spatial distribution of representative cell types (immune score, monocytes, neutrophils, endothelial cells, and fibroblasts) across 0, 24, and 96 h post-infection. Color intensity represents RCTD-normalized cell-type weight per spot, with unified color scales across timepoints for each cell type. **(B)** COMMOT spatial cell–cell communication analysis of three curated ligand–receptor pairs (Ccl2–Ccr2, Cxcl2–Cxcr2, Spp1–Cd44). Sender scores are plotted as continuous color maps, with bar plots quantifying mean sender scores at each timepoint.

To spatially resolve the cellular dynamics observed in our scRNA-seq data, we performed RCTD deconvolution on Visium spatial transcriptomics data from lung tissue sections at 0 h, 24 h, and 96 h post-infection. Deconvolution of 15 major cell types revealed distinct spatial and temporal patterns. The overall immune infiltration score—calculated as the summed RCTD weight of all immune cell types per spot—increased from 14.7% at 0 h to a peak of 22.5% at 24 h, before returning to 14.7% by 96 h, mirroring the temporal dynamics observed in scRNA-seq (**Figure 7A**, row 1).

Among immune populations, monocytes exhibited the most pronounced spatial expansion, doubling from 4.7% at 0 h to 8.2% at 24 h before declining to 3.7% at 96 h (**Figure 7A**, row 2), consistent with their role as acute-phase infiltrators. Neutrophils showed a more modest but sustained increase across the infection time course (0.7% → 1.5% → 2.1%; **Figure 7A**, row 3), reflecting persistent tissue occupancy during both the inflammatory and early resolution phases.

Structural cells displayed coordinated but divergent spatiotemporal remodeling (**Figure 7A**, rows 4–5). Endothelial cells exhibited a striking increase from 11.1% at 0 h to 20.7% at 96 h, reflecting vascular expansion and repair during the resolution phase. Fibroblasts, in contrast, showed an unexpected spatial signature: fibroblast-associated spots surged from 1.4% at 0 h to 10.3% at 24 h, before returning to 1.3% at 96 h. This spatial peak at 24 h was not driven by canonical fibroblast markers but by tissue injury and remodeling effectors—Timp1 showed the strongest gene–cell type spatial correlation in the entire dataset (r = 0.80), followed by Saa3 (r = 0.48) and Mmp3 (r = 0.39). This divergence from scRNA-seq—where fibroblast proportions progressively declined (22.3% → 9.1%) due to dilution by infiltrating immune cells—indicates that the spatial fibroblast signal captured anatomical zones of acute matrix remodeling rather than fibroblast expansion per se.

We next applied COMMOT to infer spatial cell–cell communication via optimal transport (**Figure 7B**). Analysis of three curated ligand–receptor pairs revealed dramatic temporal dynamics in chemokine signaling. Ccl2–Ccr2 communication—mediating monocyte recruitment—surged 30-fold at 24 h compared to 0 h (mean sender score: 0.220 vs. 0.007), while Cxcl2–Cxcr2 signaling—driving neutrophil chemotaxis—increased 25-fold (0.106 vs. 0.004). Both signals returned toward baseline at 96 h (Ccl2–Ccr2: 0.027; Cxcl2–Cxcr2: 0.017). In contrast, Spp1–Cd44 communication, reflecting macrophage activation and tissue remodeling, remained constitutively elevated across all timepoints (0.484 at 0 h, 1.091 at 24 h, 0.860 at 96 h).

To further characterize the immune infiltration, we performed spatial differential expression analysis comparing immune-rich versus immune-poor spots (top vs. bottom 30% by immune score). At 24 h, Cxcl3 emerged as the top differentially expressed gene in immune-rich regions (log2FC = 1.09, adjusted P < 0.05), alongside Ccl2 (log2FC = 0.77) and Ccl17 (log2FC = 0.86). These chemokines were not differentially expressed at 0 h or 96 h, indicating temporally restricted chemokine activity during peak infection.

## 4 Discussion

This study provides a comprehensive time-resolved single-cell and spatial transcriptomic atlas capturing the dynamic continuum from acute inflammatory injury to tissue repair during *Pseudomonas aeruginosa* pneumonia (0–96 h post-infection), revealing previously unrecognized cellular heterogeneity and regulatory networks that coordinate host defense and tissue repair. Our time-resolved analysis of over 41,000 cells uncovered specialized neutrophil subsets, traced the origins of inflammatory fibroblasts, characterized epithelial cell state transitions, and identified a key role for ILC2-mediated type 2 immunity and circadian regulatory programs during infection resolution.

Neutrophils are the first responders during bacterial pneumonia, yet their functional heterogeneity remains incompletely understood. Our identification of four distinct neutrophil subsets with unique temporal dynamics and transcriptional profiles extends previous findings from bulk analyses (11) and provides new insights into how neutrophil functions are specialized during infection. The N3 subset, characterized by high expression of *Ccl3*, *Il1a*, and *Gbp2*, represents a highly antimicrobial population that likely drives bacterial clearance. This finding aligns with recent single-cell studies demonstrating neutrophil heterogeneity during bacterial infection, where distinct subsets exhibit specialized effector functions and migratory properties (31, 32).

Of particular interest, the Inflammatory/Antimicrobial neutrophils subpopulation shows elevated expression of *Gbp2*. GBP2 is known to potentiate immune responses to Gram-negative bacterial infection by promoting caspase-11-dependent pyroptosis in mice (33). The co-expression of *Ccl3* and *Ccl4* suggests these neutrophils not only directly kill bacteria but also recruit additional immune cells, amplifying the antimicrobial response. Furthermore, Inflammatory/Antimicrobial neutrophils showed enrichment in NF-κB and TNF signaling pathways, indicating they are transcriptionally primed for robust inflammatory responses. This molecular signature may serve as a biomarker for identifying patients who mount effective neutrophil-mediated clearance versus those who develop persistent infections. Spatial transcriptomic analysis further validated these findings: Cxcl3 was the top differentially expressed gene in immune-rich tissue regions at 24 hpi, and COMMOT revealed a 25-fold increase in Cxcl2-Cxcr2 spatial signaling at 24 h, confirming chemokine-driven neutrophil recruitment to pulmonary infection foci.

While recent single-cell studies have begun to unravel immune cell heterogeneity during *P. aeruginosa* infection (32), the active role of structural cells-particularly fibroblasts-in orchestrating pulmonary immunity remains underexplored. A major finding of our study is the identification of Col13a1^+^ matrix fibroblasts as the primary source of inflammatory fibroblasts during acute infection. While fibroblast activation during lung injury has been described (34), the cellular origins and molecular mechanisms driving this transition remained unclear. Temporal profiling of fibroblast subpopulations revealed a progressive increase in the proportion of Col13a1+ matrix fibroblasts, which concurrently upregulated inflammatory mediators such as *Saa3*, *Lcn2*, and *Cxcl10*, suggesting their transition toward an activated, pro-inflammatory state. While RNA velocity analysis provided supportive but inconclusive directional signals, the primary evidence for this phenotypic shift stems from dynamic changes in cellular abundance and gene expression signatures across time points. The transcriptional regulatory network centered on *Stat1* and *Nfkb1* that we identified in activated fibroblasts provides mechanistic insights into how these cells coordinate inflammation. STAT1 and NF-κB are known to drive expression of interferon-stimulated genes and pro-inflammatory cytokines, respectively, but our work reveals their coordinated activity specifically within lung fibroblasts during bacterial infection. The upregulation of *Cxcl10* by activated fibroblasts is particularly important, as CXCL10 signals through CXCR3 to enhance neutrophil-mediated bacterial clearance (35), suggesting a fibroblast-neutrophil crosstalk that amplifies host defense. This finding has important implications for understanding how structural cells actively participate in immune regulation. Rather than being passive bystanders, fibroblasts emerge as key orchestrators of pulmonary immunity during infection, potentially explaining why fibrotic responses are often intertwined with chronic inflammatory conditions. The decline of activated fibroblasts after the early peak likely reflects resolution of NF-κB-driven activation and reversion toward quiescence rather than apoptotic elimination, with immune cell infiltration being the dominant factor in relative frequency changes.

Alveolar epithelial cells serve as both physical barriers and active participants in host defense (16, 36). Our characterization of three distinct AT2 cell states-homeostatic, inflammatory, and reparative-provides a framework for understanding how epithelial cells balance defense and repair functions during infection. The inflammatory AT2_2 state, which dominated during bacterial clearance, upregulated genes involved in cell killing and defense responses, demonstrating that epithelial cells directly contribute to antimicrobial immunity. Perhaps most intriguingly, we discovered a circadian regulatory program involving *Dbp* and *Per3* that emerges specifically during the resolution phase (96 hpi). The specific upregulation of Dbp in AT2 cells and other structural cells during resolution suggests circadian genes may coordinate tissue repair programs (37). This finding is novel in the context of bacterial pneumonia and raises important questions about how circadian disruption (common in critically ill patients) might affect recovery from infection. Recent studies have highlighted the importance of circadian rhythms in immune function (38), but our work is among the first to identify circadian gene expression specifically during infection resolution in the lung. The predicted interaction between DBP and PER3 suggests a functional circadian clock may be re-established during recovery, potentially optimizing tissue repair processes. This has translational implications, as timing of therapeutic interventions to align with circadian rhythms could potentially enhance recovery in patients with severe pneumonia.

Our analysis revealed a functional dichotomy among alveolar macrophages during *P. aeruginosa* infection. AM_2 exhibited a pronounced pro-inflammatory program—chemokine production, complement activation, and interferon signaling—consistent with a host-defense macrophage driving bacterial clearance. The near-universal expression of *Irg1/Acod1* in AM_2 (82.2% vs. 1.3% in AM_1) suggests itaconate-mediated antibacterial activity, while upregulation of *Lcn2* and *Fth1* indicates nutritional immunity via iron sequestration. In contrast, AM_1 retained tissue-resident functions—lipid metabolism, M2-like anti-inflammatory signatures, and negative immune regulation—likely contributing to surfactant homeostasis and inflammation resolution. The coexistence of these subsets mirrors the M1/M2 paradigm and aligns with reports of pathogen-driven AM diversification in other pulmonary infections, suggesting that the AM_1/AM_2 balance may determine infection outcome. Future studies using lineage tracing and time-course analysis are needed to resolve the developmental relationship between these populations and validate functional predictions at the protein level. Spatially, IM_2 macrophages were exclusively detected at 24 hpi within immune-infiltrated regions, and COMMOT analysis revealed a 30-fold increase in Ccl2-Ccr2 communication intensity at 24 h, providing direct spatial evidence for chemokine-mediated monocyte recruitment during acute P. aeruginosa infection.

Group 2 innate lymphoid cells have emerged as critical regulators of tissue homeostasis and repair (39), yet their role in *P. aeruginosa* pneumonia remained poorly defined. Our discovery that epithelial-derived IL-33 activates ILC2s via the ST2 receptor during acute infection (24 hpi) provides a mechanistic link between early tissue damage and the initiation of type 2 immune responses. Notably, the peak activation of NF-κB in ILC2s at 24 hours post-infection indicates that these cells are rapidly engaged in the acute inflammatory phase, rather than solely participating in later resolution processes. This IL-33/ST2 axis likely stimulates ILC2s to produce type 2 cytokines that modulate excessive inflammation and initiate protective repair programs concurrently with ongoing bacterial clearance. Previous studies have implicated ILC3s, rather than ILC2s, in the response to *P. aeruginosa* infection, primarily through IL-17 production (39, 40). However, our findings reveal that ILC2s and ILC3s are concurrently active during acute infection, suggesting a more complex and coordinated innate lymphoid cell response than previously appreciated. Rather than strict temporal specialization (ILC3s early, ILC2s late), our data support a model in which ILC2s are rapidly mobilized to temper inflammation and promote epithelial resilience while bacterial clearance is still ongoing. The transient activation of NF-κB in ILC2s that we observed likely represents a tightly regulated feedback mechanism to prevent excessive type 2 responses that could compromise antimicrobial immunity. This finding is consistent with studies showing that IL-33 administration can improve outcomes in bacterial pneumonia through effects on both neutrophils and monocytes (41), and our work now identifies ILC2s as a key IL-33-responsive population that contributes to immune balance during the acute phase of infection.

The integration of spatial transcriptomics with time-resolved scRNA-seq provided three orthogonal insights that dissociation-based profiling alone could not resolve. First, RCTD deconvolution revealed that immune infiltration peaked locally at 22.5% of tissue area at 24 h—a spatial metric that is independent of global cell-count dilution artifacts inherent to scRNA-seq. Second, COMMOT analysis pinpointed the precise anatomical foci of chemokine activity, showing that Ccl2–Ccr2 and Cxcl2–Cxcr2 signaling surged 30-fold and 25-fold, respectively, at 24 h before returning to baseline—a temporal-spatial dynamic invisible to non-spatial ligand–receptor inference. Third, and most unexpectedly, spatial gene–cell type correlation analysis exposed a critical distinction between cell-type abundance and activation state: the 24 h fibroblast spatial signal (10.3% tissue area) was driven not by fibroblast-lineage markers but by acute injury effectors Timp1 (r = 0.80), Saa3 (r = 0.48), and Mmp3 (r = 0.39). This demonstrates that RCTD, when applied to cell types sharing injury-response transcriptional programs with other populations, can conflate microenvironmental activation with cell-type presence—a cautionary finding with implications for spatial deconvolution studies across tissues.

Several limitations of our spatial analysis warrant consideration. The Visium platform captures spots of 55 μm diameter, each containing multiple cells, and our RCTD deconvolution—while robust at the major cell-type level—cannot resolve individual cells within a spot. The fibroblast Timp1-driven signal at 24 h exemplifies this limitation: we interpret it as tissue remodeling activity rather than fibroblast expansion, but single-cell resolution spatial technologies would be required to definitively separate cell identity from microenvironmental gene expression. Additionally, our COMMOT analysis was restricted to three curated ligand–receptor pairs; a comprehensive spatial ligand–receptor screen across all expressed pairs may reveal additional communication axes. Finally, spatial transcriptomics was performed at three timepoints with one section each; biological replication across multiple tissue sections per timepoint would strengthen the generalizability of the spatial patterns we report.

This study has several limitations that should be acknowledged. First, while we identified ILC2 activation during the resolution phase, causal evidence for their functional contribution to tissue repair remains indirect. Second, while our time-course provides good temporal resolution, earlier timepoints (e.g., 3–6 hpi) could reveal additional insights into the initial host response. Third, our study used a single *P. aeruginosa* strain (PAO1); responses to other strains with different virulence profiles warrant investigation.

Future work should focus on validating the functional roles of the cell populations we identified through genetic and pharmacologic approaches. For example, selective depletion of N3 neutrophils or activated fibroblasts could confirm their contributions to bacterial clearance and inflammation. Additionally, single-cell analyses of human samples from patients with *P. aeruginosa* pneumonia would be valuable for translating our findings to the clinical setting.

The role of circadian genes in infection resolution deserves further investigation. Experiments using circadian-disrupted mice or pharmacologic modulation of circadian pathways could determine whether circadian regulation is causally linked to recovery outcomes. Finally, therapeutic studies targeting the IL-33/ST2/ILC2 axis at different timepoints could establish optimal windows for intervention to promote resolution without compromising bacterial clearance.

In conclusion, our integrated time-resolved single-cell and spatial transcriptomic analysis reveals the dynamic cellular landscape of the lung during acute *P. aeruginosa* infection and provides a foundation for developing targeted therapies that enhance bacterial clearance while promoting tissue repair. By combining single-cell resolution of transcriptional states with spatial mapping of cellular neighborhoods, we uncovered not only specialized neutrophil subsets, inflammatory fibroblast origins, epithelial state transitions, and ILC2 activation pathways, but also their precise anatomical localization and spatial relationships within the infected lung. This spatially informed cellular atlas advances our understanding of host-pathogen interactions and highlights multiple potential targets for host-directed therapies.

## Data Availability

The datasets presented in this study can be found in online repositories. The names of the repository/repositories and accession number(s) can be found below: NCBI Gene Expression Omnibus (GEO), accession number GSE345596 (https://www.ncbi.nlm.nih.gov/geo/query/acc.cgi?acc=GSE345596). Scripts for the analyses are available from the corresponding authors upon request.
