# Supplementary material for: Dynamic orchestration of structural and immune cells in acute Pseudomonas aeruginosa pneumonia revealed by time-resolved single-cell and spatial transcriptomics
Source: Front Immunol. 2026 Sep 8;17:1874223. doi: 10.3389/fimmu.2026.1874223 (PMC13593461; doi:10.3389/fimmu.2026.1874223)
Supplement: Supplementary file 1 [file SupplementaryFile1.docx]

Supplementary Material

**Supplementary Figure 1:** **Apoptosis and proliferation dynamics of fibroblast subpopulations during *P. aeruginosa* infection.**

Supplementary Figure 1A: Apoptosis scores of total fibroblasts across five timepoints (0, 12, 24, 48, and 96 h post-infection), showing a biphasic apoptotic signature peaking at 12 hpi.

Supplementary Figure 1B: Survival scores comparing activated fibroblasts versus homeostatic fibroblasts across timepoints.

Supplementary Figure 1C: Apoptosis scores comparing activated fibroblasts versus homeostatic fibroblasts across timepoints.

Supplementary Figure 2: Spatial localization of activated fibroblasts inferred by RCTD deconvolution of spatial transcriptomics data. Color intensity reflects the posterior probability of activated fibroblast presence at each spot, with deeper red indicating higher confidence in their assignment.
